# Intracanal microbiome profiles of two apical periodontitis cases in one patient: A comparison with saliva and plaque profiles

**DOI:** 10.1002/cre2.862

**Published:** 2024-03-03

**Authors:** Keiko Yamaki, Toru Tamahara, Jumpei Washio, Takuichi Sato, Ritsuko Shimizu, Satoru Yamada

**Affiliations:** ^1^ Division of Periodontology and Endodontology, Graduate School of Dentistry Tohoku University Sendai Japan; ^2^ Tohoku Medical Megabank Organization Tohoku University Sendai Japan; ^3^ Division of Oral Ecology and Biochemistry, Graduate School of Dentistry Tohoku University Sendai Japan; ^4^ Division of Clinical Chemistry, Graduate School of Health Sciences Niigata University Niigata Japan; ^5^ Department of Molecular Hematology, Graduate School of Medicine Tohoku University Sendai Japan

**Keywords:** 16S rRNA gene analysis, apical periodontitis, endodontic microbiome, root canal

## Abstract

**Objectives:**

To determine the characteristics of the endodontic microbiome.

**Material and Methods:**

Saliva, plaque, and infected root canal wall dentin of two teeth suffering from apical periodontitis were harvested from a 58‐year‐old man. Bacterial DNA was extracted from each sample, and 16S rRNA gene analysis targeting the V3–V4 region was conducted on the Illumina MiSeq platform using QIIME2. The functional potential of the microbiomes was inferred using PICRUSt2.

**Results:**

The four microbiomes were different in structure and membership, yet the nine most abundant metabolic pathways were common among them. The two endodontic microbiomes were more anaerobic, rich in Firmicutes, and scarce in Actinobacteriota and Proteobacteria, compared with saliva and plaque microbiomes. Their profiles were dissimilar despite their clinical and radiographic similarities.

**Conclusions:**

The endodontic microbiomes were anaerobic, rich in Firmicutes, scarce in Actinobacteriota and Proteobacteria, and considerably varied within an individual.

## 1 INTRODUCTION

Apical periodontitis is an inflammatory reaction caused by oral bacteria colonizing the root canal (RC) system (Kakehashi et al., 1965). Endodontic infections have a polymicrobial nature, and community profiling studies have revealed that the bacterial composition of the endodontic microbiome differs consistently among individuals suffering from the same disease (Siqueira & Rôças, 2009b). The high interindividual variability observed for samples from the same clinical disease indicates that different bacterial community compositions can result in similar disease outcomes. This indicates that apical periodontitis has a heterogeneous etiology, where multiple bacterial combinations can play a role in disease causation. Therefore, no single species can be considered as the main endodontic pathogen, and the “community as pathogen” concept in the etiology of apical periodontitis is now widely embraced (Siqueira & Rôças, 2005, 2009a, 2013). Nevertheless, the reason for such large differences in bacterial species found in the same disease remains of interest.

The oral microbiome comprises more than 700 species of bacteria and is the second largest and most diverse microbiome in the human body after the gut (Dewhirst et al., 2010; Paster et al., 2006; Verma et al., 2018). Because one person can harbor approximately 100–200 taxa in his or her oral cavity (Paster et al., 2006), a substantial interindividual diversity exists in the oral microbiome, which might be somewhat responsible for the variability of the endodontic microbiome. Many oral bacteria have a known site‐specific habitat within the human oral cavity (Aas et al., 2005; Paster et al., 2006; Segata et al., 2012; Simón‐Soro et al., 2013), so that the bacterial composition at an oral niche is distinctive and unique to the site. Saliva, which was once thought to represent all the bacteria found on oral surfaces, is strongly biased toward the tongue and palate communities, and its microbial profile is different from that of dental plaque.

The clinical and structural conditions of the involved teeth may also affect the environment within their RCs, wielding strong selective pressure against bacterial growth therein. Teeth without a history of treatment (primary infections) tend to show highly diverse bacterial profiles, while canal‐treated teeth (secondary/persistent infections) have a decreased diversity because of their harsh environment for bacteria to survive and flourish, with restricted space and nutrient deprivation brought by the previous procedures (Korona‐Glowniak et al., 2021; Siqueira & Rôças, 2009b). Furthermore, the efficacy of coronal and apical seal can alter the bacterial population in the canal. The coronal leakage resulting from inadequate restorations leads to salivary contamination, and the absence of the apical seal allows numerous nutrients to seep into the canal via blood vessels. Previous studies have revealed that the success rate is the highest when good root filling is achieved in combination with good restoration, showing the critical importance of the coronal seal (Ray & Trope, 1995; Vimal & Poonam, 1996).

To the best of our knowledge, there are few reports on the endodontic microbiome that also consider saliva and/or plaque profiling in the same subject. We encountered a patient whose two endodontically treated incisors had apical periodontitis, and through 16S rRNA gene analysis on the Illumina MiSeq platform by targeting the V3–V4 hypervariable region, we were able to profile these two intracanal microbiomes along with his saliva and plaque. The aim of this study is to determine the characteristics of the endodontic microbiome by comparing the two intracanal microbiomes with the microbial profiles of saliva and plaque. We also compared the two intracanal microbiomes to reveal the intraindividual variability of the endodontic microbiome.

## 2 MATERIALS AND METHODS

The patient was a 58‐year‐old man who visited the Tohoku University Hospital for multidisciplinary dental treatment. He was a nonsmoker and was taking antihypertensive medications. Two mandibular incisors, 42 and 31 according to the FDI two‐digit system, were sensitive to apical palpation and horizontal percussion, respectively. Both teeth had been canal‐obturated short of the apex and were accompanied by apical radiolucency. Neither tooth had a sinus tract. From these findings, the two teeth were diagnosed with apical periodontitis in need of RC therapy. Informed consent for sample collection was obtained in a written form, and the prosthetic crowns were removed and replaced with temporary prostheses in advance to enable aseptic endodontic treatment.

Sample collection was performed on the first visit for RC treatment. To assess the oral environment at the time of dental treatment, no restrictions had been placed on the patient's behavior as to eating and tooth brushing before sample collection. The patient was instructed to sit and take time to collect his resting saliva in a 50 mL tube, from which 1 mL was transferred into a microtube using a pipette. Supragingival plaque was collected from two maxillary premolars, 14 and 15, by gently scaling their buccal surfaces with a Gracey‐type curette. The amassed plaque was suspended in a microtube containing saline. RC dentin was collected using hand files. Endodontic sampling and treatment were performed under strict aseptic conditions by an experienced endodontist. Each tooth was isolated by a rubber dam and disinfected with iodine tincture and 70% ethanol. A sterile hand‐file #15 reamer was introduced into the canal through the access cavity, and a vigorous filing motion was applied to produce dentin shavings. The file was then pulled out from the canal, and its dentin‐smeared blade portion was cut off using a sterile wire cutter and placed in a microtube containing saline to serve as an RC sample. This procedure was repeated several times with K‐ and/or H‐type files of progressively larger sizes to ensure dentin harvest. The teeth were subjected to conventional RC treatment after sampling. The samples were transported to the Tohoku Medical Megabank laboratory and stored at −80°C until analysis. The study protocol was approved by the Ethics Committee of the Tohoku University Graduate School of Dentistry.

DNA extraction was performed using the QIAamp UCP Pathogen Mini Kit (Qiagen), and the V3–V4 region of the 16S rRNA gene was amplified, barcoded, and sequenced on the Illumina MiSeq platform following the manufacturer's instructions. The primers used for PCR amplification were V3V4f_MIX and V3V4r_MIX (Table S1), provided by the Bioengineering Lab. Co., Ltd.

The amplicon sequence data were analyzed using QIIME2 (Bolyen et al., 2019) version 2020‐8, with SILVA (Quast et al., 2013) as the reference database. All paired reads were trimmed to remove primer sequences and low‐quality sequence data. Potential amplicon sequencing errors were corrected using DADA2 (Callahan et al., 2016) to produce an amplicon sequence variant (ASV) data set. The resultant ASVs were aligned using MAFFT (Katoh & Standley, 2013) and then a phylogenetic tree was constructed using FastTree2 (Price et al., 2010). The α‐diversity metrics were estimated from the processed ASV data sets. Each ASV was identified using a Naïve Bayes classifier trained on 16S rRNA gene sequences from the SILVA database (release 138, 2019) and assigned at the species level. Principal coordinate analysis (PCoA) and other multivariate analyses were performed using Python and R scripts. The functional potential of the microbiomes was inferred using PICRUSt2, which calculated MetaCyc pathway abundances through structured mappings of EC gene families to pathways (Caspi et al., 2018, 2020; Douglas et al., 2020).

Due to the small sample size, statistical analysis was not performed.

## 3 RESULTS

**Figure 1:**
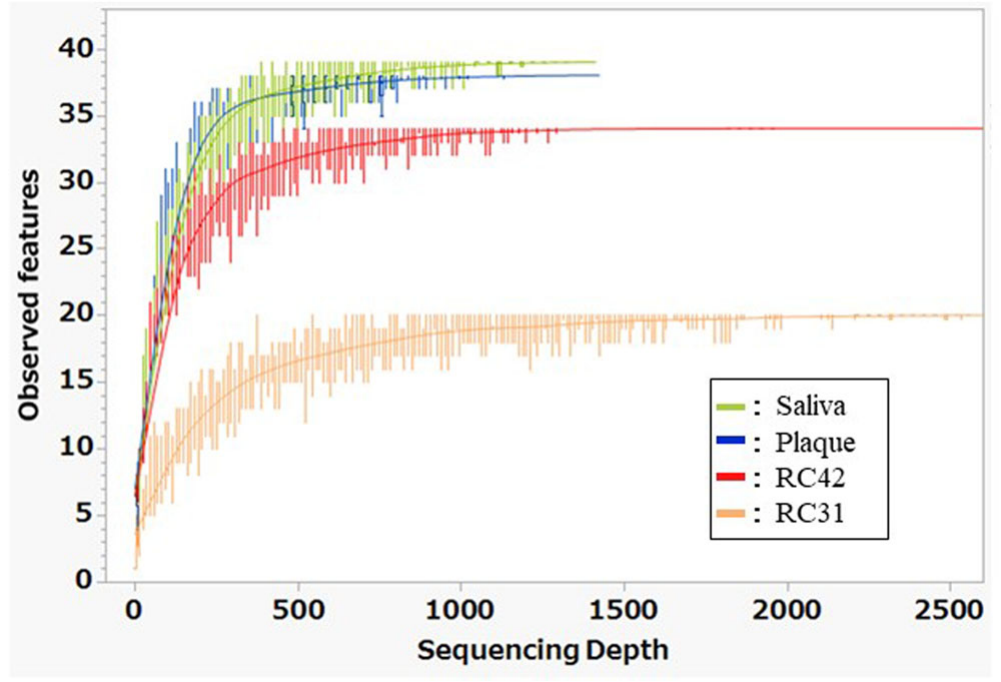
Rarefaction curves for each sample. The plateaus showed that the bacterial richness in the samples was completely revealed by the numbers of sequences analyzed in this study. RC42, root canal sample obtained from Tooth 42; RC31, root canal sample obtained from Tooth 31.

A total of 8908 sequence reads were obtained from the four samples after quality control. Each sample yielded >1000 reads and rarefaction analysis showed that the bacterial richness of the samples was completely revealed by these sequences (Figure 1). Table 1 lists the α‐diversity metrics estimated from the ASV data sets.

**Table 1:** Alpha diversity metrics.

Sample	Reads	Observed features	Shannon entropy	Faith's PD
Saliva	1219	39	4.171	4.000
Plaque	1447	38	4.406	3.967
RC42	2816	34	3.981	3.787
RC31	3426	20	2.346	3.169

Abbreviations: PD, phylogenetic diversity; RC42, root canal sample obtained from tooth 42; RC31, root canal sample obtained from Tooth 31.

**Figure 2:**
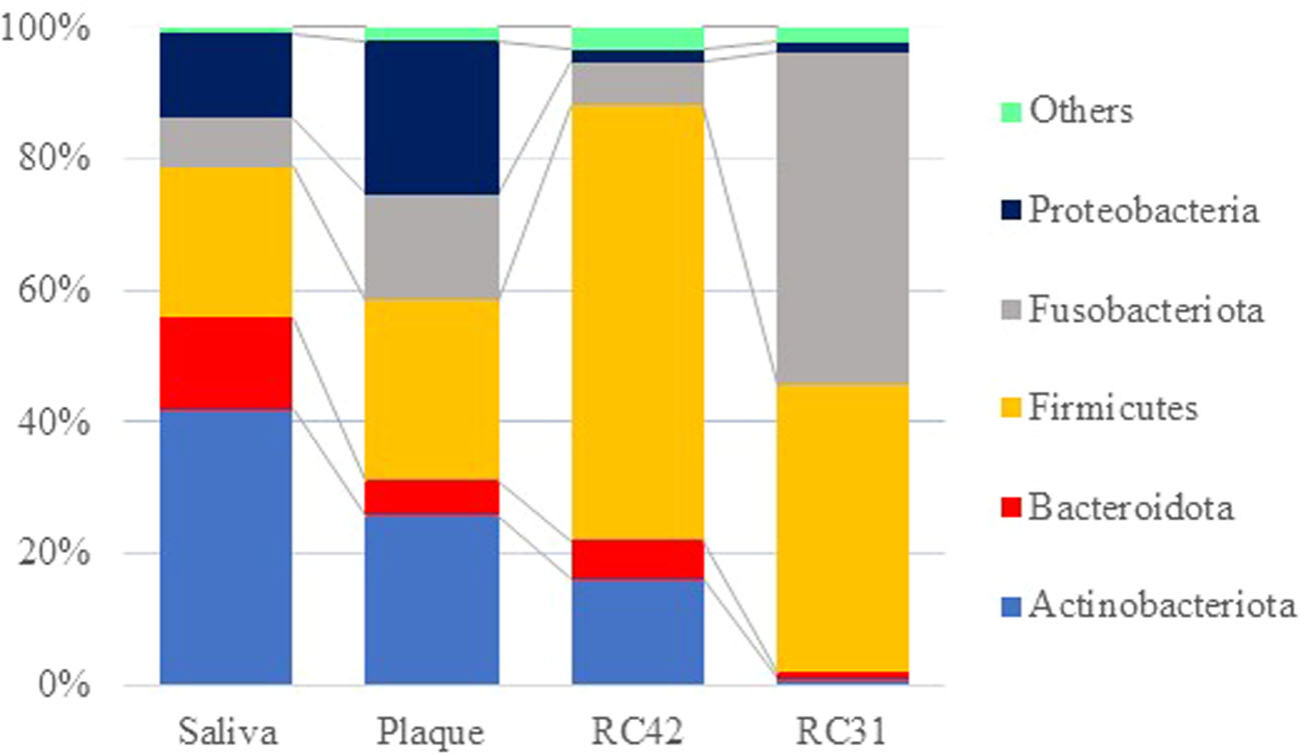
Taxonomic assignment at the phylum level. The root canal (RC) samples obtained from Tooth 42 and Tooth 31 were both rich in Firmicutes and scarce in Actinobacteriota and Proteobacteria.

**Figure 3:**
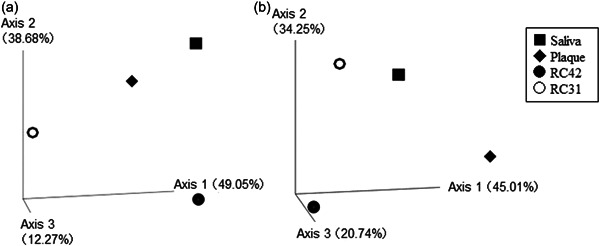
Three‐dimensional principal coordinate analysis (PCoA) plots based on (a) weighted and (b) unweighted UniFrac distances, showing the dissimilarity among the samples. RC42, root canal sample obtained from Tooth 42; RC31, root canal sample obtained from Tooth 31.

Taxonomic data revealed that the microbial compositions of the four samples were different, not only at the genus/species level but also at the higher phylum level. As shown in Figure 2, both RC samples were rich in Firmicutes and scarce in Actinobacteriota and Proteobacteria. In contrast, the latter two phyla were more abundant and cumulatively constituted 54.7% and 49.1% of the saliva and plaque microbiomes, respectively. The phylum Campilobacteriota was classified into the category “Others” in Figure 2, as its proportion was less than 4% in all samples (range 0.74%‒3.41%). The ASV data sets recovered from the samples were clustered into 48 groups, that is, 44 genera and another four that failed to reach the genus level (Table S2). PCoA based on weighted and unweighted UniFrac distances indicated that the four microbiomes were phylogenetically different (Figure 3).

The saliva sample contained 31 taxa, of which 12 belonged to Firmicutes. This was the largest number observed among the four samples, despite its lowest relative abundance of 23.1%. Additionally, the ratio of Actinobacteriota (41.8%) was the highest among the samples. This was due to the presence of *Rothia*, which constituted 37% of the total reads. The five most frequently observed genera in the saliva were *Rothia, Neisseria, Prevotella, Stomatobaculum*, and *Streptococcus*. Anaerobes comprised 50.0% of the salivary microbiome.

The plaque sample yielded 28 taxa. The phyla Firmicutes, Actinobacteriota, and Proteobacteria comprised approximately 25% each, and the ratio of Proteobacteria at 23.4% was the highest among the four samples. The five most abundant genera in plaque were *Neisseria, Streptococcus, Actinomyces, Fusobacterium*, and *Leptotrichia*. The genus *Streptococcus* was identified in all four samples, and its relative abundance at 20.6% in the plaque was the highest. While *Rothia* was the predominant member of Actinobacteriota in saliva, it was overwhelmed by *Actinomyces* in plaque. The proportion of anaerobes in the plaque sample was 67.6%.

Tooth 42 harbored 29 phylotypes in its canal, with Firmicutes representing 66.2% of its community. *Dialister, Anaeroglobus*, and *Veillonella* were the main members of its Firmicutes, each with a relative abundance of >14%. These three genera were either absent or scarce in the saliva and plaque samples. The genera *Rothia, Fusobacterium*, and *Prevotella* were also substantially abundant (>5%) in its canal. The proportion of anaerobes in RC42 was 89.0%.

Although a total of 17 phylotypes were detected, the RC of Tooth 31 was dominated by two obligate anaerobes, *Pseudoramibacter* (41.5%) and *Fusobacterium* (50.7%). Each of the remaining 15 taxa was less than 1% except for *Campylobacter gracilis* (2.1%), and the community richness indices of RC31 were the lowest (Table 1). Anaerobes comprised 98.0% of the population.

**Figure 4:**
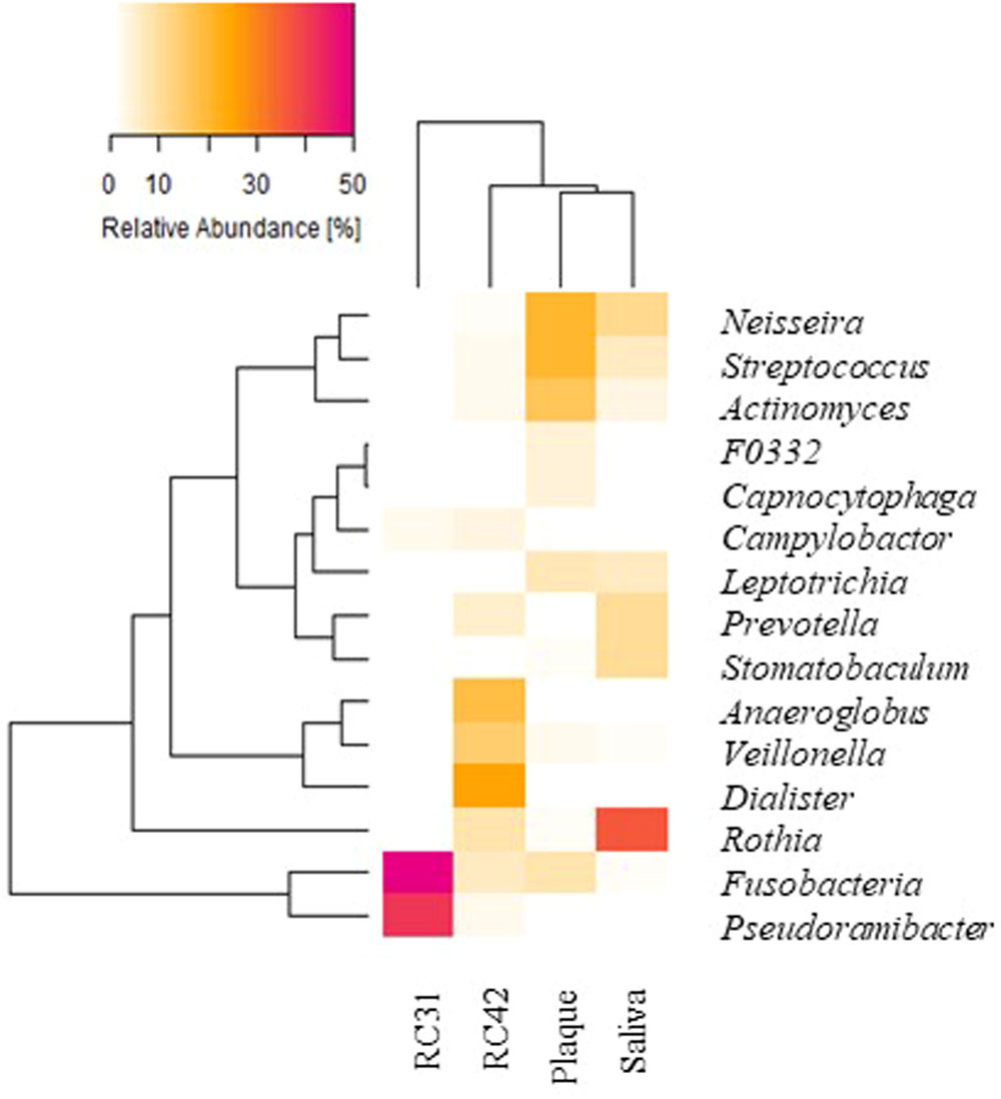
Heatmap showing the prevalence and distribution of the 15 main genera among the samples. RC42, root canal sample obtained from Tooth 42; RC31, root canal sample obtained from Tooth 31.

**Figure 5:**
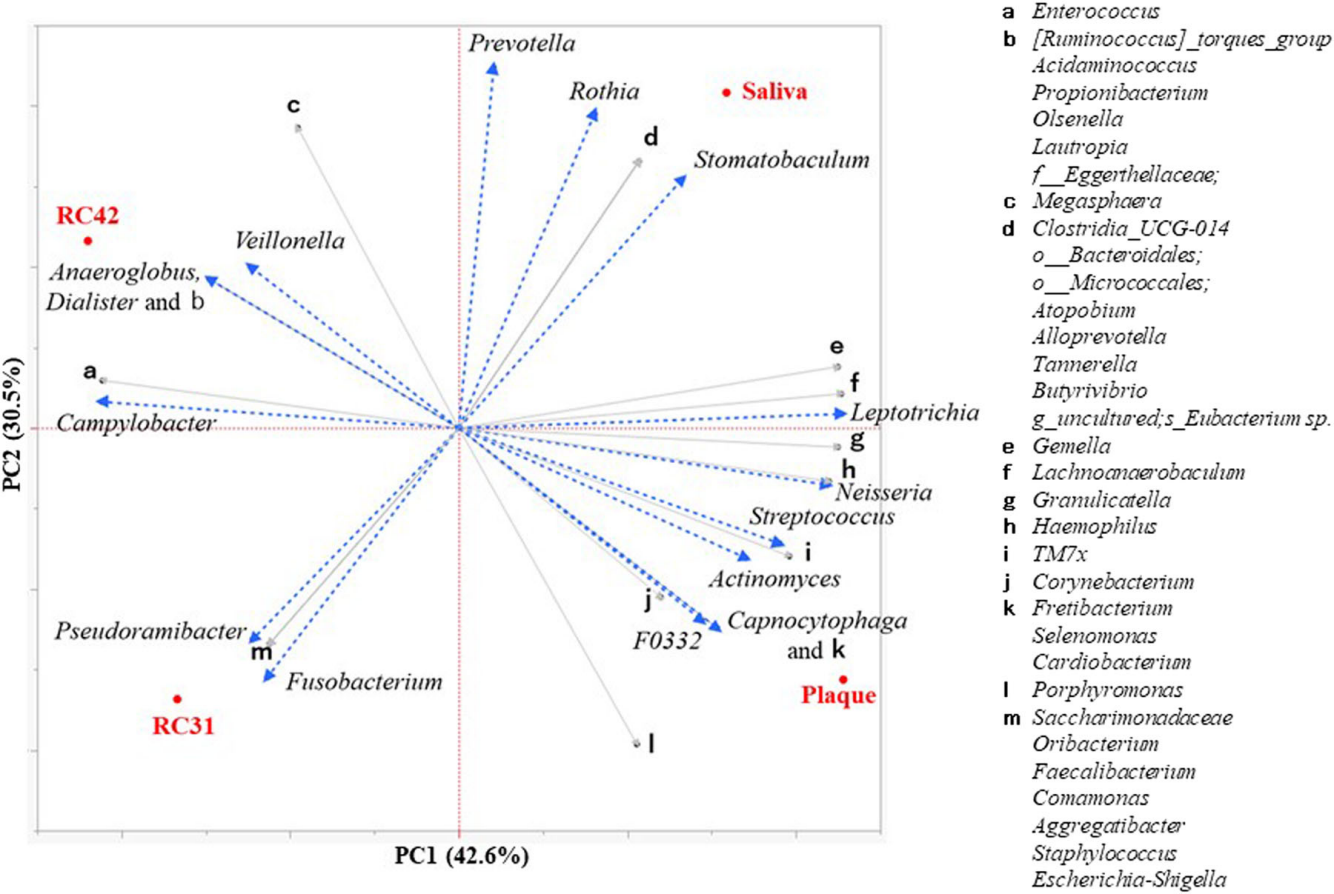
Biplot of two‐dimensional principal component analysis (PCA) and factor loadings revealed the difference in bacterial membership among the samples. Dotted arrows in blue indicate the factor loadings of the 15 main genera, and gray arrows indicate those of the other phylotypes. RC42, root canal sample obtained from Tooth 42; RC31, root canal sample obtained from Tooth 31.

The six most abundant genera in each sample totaled 15 genera, which collectively accounted for 89.3%, 88.0%, 91.4%, and 97.3% of the population in saliva, plaque, RC42, and RC31, respectively. The relative abundance and prevalence of these representative genera are listed in Table 2 and visualized in Figure 4. *Rothia*, *Streptococcus*, *Fusobacterium*, *Neisseria*, and *Campylobacter* were found in all four samples, with variable abundances. Principal component analysis (PCA) at the genus level revealed that each sample had a distinct microbial structure. As shown in Figure 5, the genera *Leptotrichia, Neisseria*, and *Streptococcus* had large positive loads on principal component 1 (PC1) and the genus *Campylobacter* had a large negative load on PC1. The genera *Prevotella* and *Rothia* had large positive loads on PC2 (Table S3).

**Table 2:** Prevalence and distribution of the 15 main genera in percentile.

Phylum/genus	Saliva	Plaque	RC42	RC31
Actinobateriota	41.8	25.7	16.2	0.9
*Actinomyces*	3.70	16.72	2.90	
*Rothia*	37.00	1.24	8.80	0.88
*F0332*		4.08	0.36	
Bacteridota	13.9	5.3	5.8	0.9
*Capnocytophaga*		4.08		
*Prevottela*	10.25		5.79	0.32
Firmicutes	23.1	27.4	66.2	43.8
*Anaeroglobus*			18.22	
*Dialister*			25.18	
*Pseudoramibacter*			2.24	41.48
*Stomatobaculum*	10.17	1.38		
*Streptococcus*	6.89	20.59	2.73	0.85
*Veillonella*	1.6	2.07	14.35	
Fusobacteriota	7.3	16.2	6.5	50.7
*Fusobacterium*	1.23	8.71	6.50	50.73
*Leptotorichia*	6.07	7.53		
Proteobacteria	12.9	23.4	2.0	1.5
*Neisseria*	11.65	20.73	1.10	0.93
Campilobacterota	0.7	0.9	3.4	2.1
*Campylobacter*	0.74	0.90	3.41	2.13

Abbreviations: RC42, root canal sample obtained from Tooth 42; RC31, root canal sample obtained from Tooth 31.

**Figure 6:**
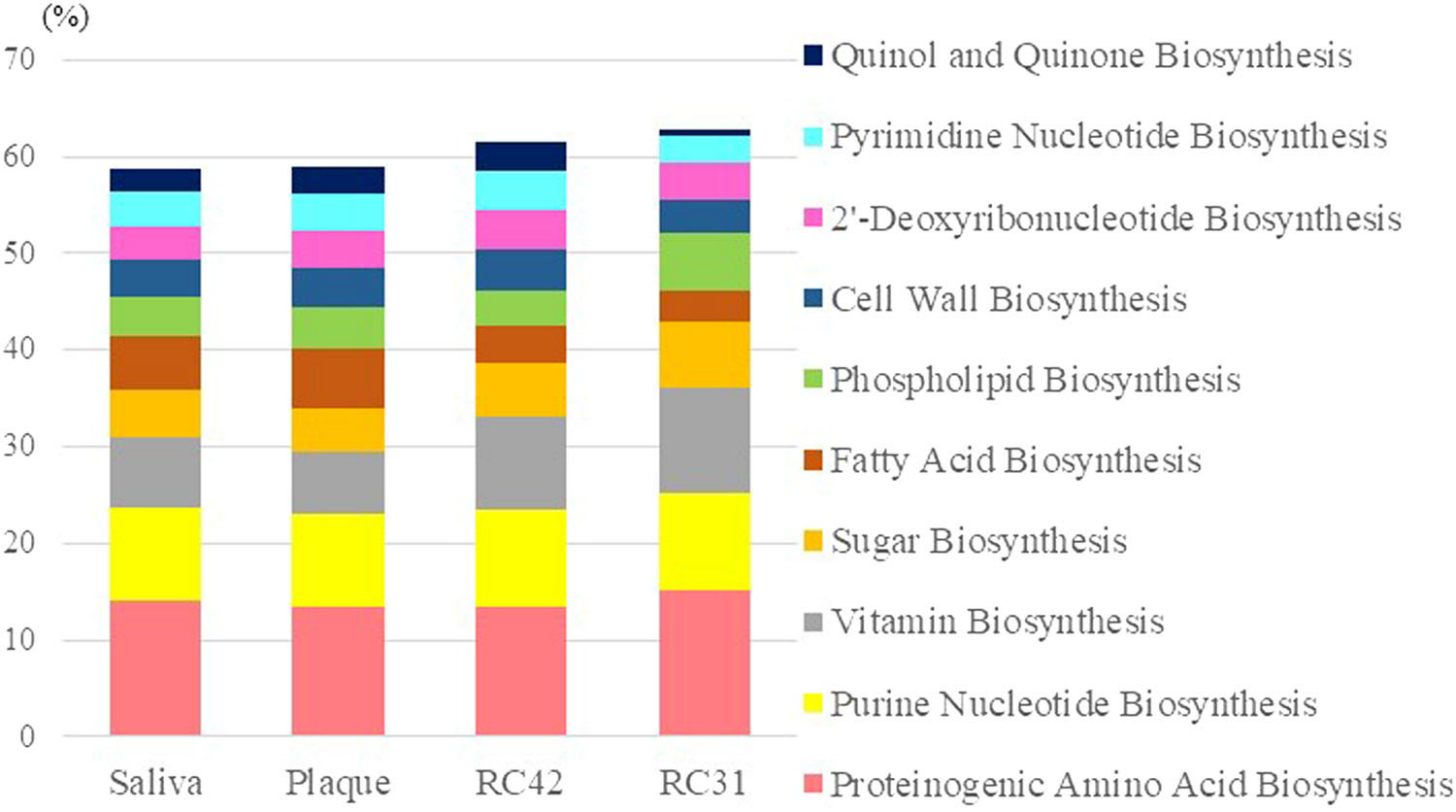
Relative abundances of the 10 major pathways. RC42, root canal sample obtained from Tooth 42; RC31, root canal sample obtained from Tooth 31.

The functional potential of each microbiome was speculated using PICRUSt2. At Level 3, the 10 most abundant MetaCyc pathways in saliva also ranked within the top 10 in other samples except for quinol and quinone biosynthesis, which ranked 35th in RC31. These 10 pathways totaled approximately 60% in each sample (Figure 6). The common top nine pathways were proteinogenic amino acid biosynthesis, purine nucleotide biosynthesis, vitamin biosynthesis, fatty acid biosynthesis, sugar biosynthesis, phospholipid synthesis, cell wall synthesis, pyrimidine nucleotide biosynthesis, and 2′‐deoxyribonucleotide biosynthesis. Most maintained similar proportions across the samples, for example, purine nucleotide biosynthesis was 9.75%–10.0%, and 2′‐deoxyribonucleotide biosynthesis was 3.4%–4.0%. However, in RC samples, the level of vitamin biosynthesis was higher and that of fatty acid biosynthesis was lower than those in saliva and plaque. Moreover, the abundance of phospholipid biosynthesis was higher in RC31 than in the other three samples.

**Figure 7:**
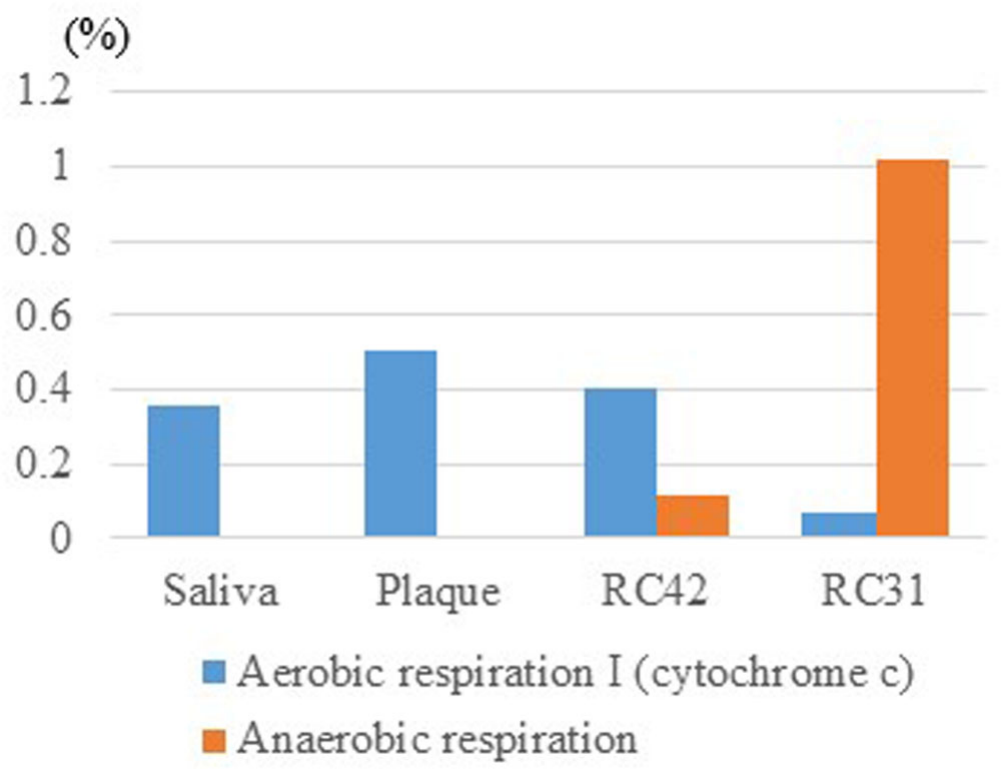
Relative abundances of aerobic and anaerobic respiration. RC42, root canal sample obtained from Tooth 42; RC31, root canal sample obtained from Tooth 31.

Of the 159 pathways listed in Level 3, anaerobic respiration ranked 21st in RC31 despite its low rankings in the other three samples, leading to a stark difference in the respiration pattern among the samples (Figure 7). Other characteristics of RC31 were enhancement of cobyrinate a,c‐diamide biosynthesis, isopropanol biosynthesis, and (S) propane‐1,2‐diol degradation.

**Figure 8:**
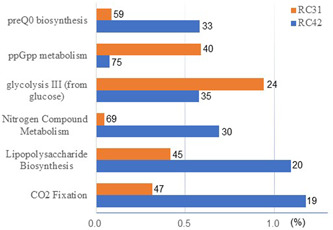
Pathways with considerable differences in relative abundance between the two root canal (RC) samples. Numbers beside the bars indicate their ranks within each sample. RC42, root canal sample obtained from Tooth 42; RC31, root canal sample obtained from Tooth 31.

Pathways that showed relatively large differences (Δ*d* in abundance >2 × standard deviation, and Δ*d* in rank >10) between RC42 and RC31 included CO_2_ fixation, lipopolysaccharide biosynthesis, nitrogen compound metabolism, glycolysis III, ppGpp metabolism, and preQ_0_ biosynthesis. RC42 was more abundant in four of these six pathways, and RC31 outperformed RC42 only in glycolysis Ⅲ and ppGpp metabolism (Figure 8).

## 4 DISCUSSION

Three types of oral specimens—saliva, plaque, and infected RC wall dentin—were collected from a patient with apical periodontitis, and their microbial structures were investigated via 16S rRNA sequence analysis targeting the V3–V4 hypervariable region. Protocols targeting the V3–V4 region are widely used in oral microbiome research which yield profiles representative of diverse communities at the genus level (Wade & Prosdocimi, 2020).

Because the blade portion of a hand file could carry only a limited amount of dentin, multiple files were employed to harvest as many dentin shelves as possible. Four files were used for RC42 and five were used for RC31, with #25 as the final and largest file size in both cases. Nevertheless, bacterial loads in the RC samples were so meager that in a preliminary test, we failed to obtain enough DNA with our conventional protocol, which uses a DNeasy PowerSoil Pro Kit (Qiagen) for DNA extraction. Therefore, in this study, we used a QIAamp UCP Pathogen Mini Kit for DNA extraction. This kit enables efficient cell lysis of both Gram‐positive and Gram‐negative bacteria through physical and chemical cell wall disruption by beads and protein kinase K, respectively, and is known to be suitable for a small number of bacteria in amniotic fluid and blood (Lim et al., 2022; Urushiyama et al., 2017). Thus, we could extract a sufficient amount of bacterial DNA from the RC samples, as well as from saliva and plaque and proceeded to 16S analysis.

A total of 8908 sequence reads were obtained from the four samples, and their taxonomic data were clustered into 48 phylotypes, of which 44 could be assigned to the genus level. The structural difference in their microbiomes was evident at the phylum level, and the difference in their membership was confirmed by PCA at the genus level. Furthermore, they were phylogenetically different according to the PCoA based on UniFrac distances. Previous studies have confirmed that saliva cannot substitute for the bacterial composition of the diseased oral niche (Aas et al., 2005; Paster et al., 2006; Segata et al., 2012; Simón‐Soro et al., 2013). In this study, the number of taxa found in saliva was the highest yet comparable to those in plaque and RC42, and certain species were found only in plaque or only in RC samples. These findings indicated that saliva cannot serve as a reservoir for oral bacteria.

The six most abundant genera in each sample totaled 15 genera, which collectively made up 89.3%, 88.0%, 91.4%, and 97.3% of the relative abundance in saliva, plaque, RC42, and RC31, respectively. These genera were, in decreasing order of mean relative abundance: *Fusobacterium, Rothia, Pseudoramibacter, Neisseria, Streptococcus, Dialister, Actinomyces, Anaeroglobus, Veillonella, Prevotella, Leptotrichia, Stomatobaculum, Campylobacter, F0332*, and *Capnocytophaga*. Of these 15 representative genera, RC42 contained 12, followed by plaque with 11, saliva with 10, and RC31 with seven genera. Most are well‐known major components of saliva and plaque (Chattopadhyay et al., 2019; Diaz et al., 2016; Lee et al., 2021; Segata et al., 2012) and have also been recovered from RCs in many reports (Korona‐Glowniak et al., 2021; Martinho et al., 2021; Narayanan & Vaishnavi, 2010; Özok et al., 2012; Persoon et al., 2017; Tawfik et al., 2018; Vengerfeldt et al., 2014; Zahran et al., 2021). *Dialister, Pseudoramibacter, Streptococcus, Veillonella, Actinomyces, Fusobacterium, Campylobacter*, and *Prevotella* are among the common representative phylotypes found in endodontic infections and are considered candidate endodontic pathogens (Siqueira & Rôças, 2009b). Özok et al. (2012) profiled the bacterial communities of 23 teeth with apical periodontitis and reported 50 prevalent taxa, including *Actinomyces, Streptococcus, Pseudoramibacter, Veillonella, Fusobacterium, Neisseria, Leptotrichia, Dialister*, and *Rothia*. In this study, *Fusobacterium, Rothia, Neisseria, Streptococcus*, and *Campylobacter* were found in all samples, whereas *Pseudoramibacter, Dialister*, and *Anaeroglobus* were exclusive to the RC samples. Although *Leptotrichia* and *Capnocytophaga* were absent in our RC samples, they could also be frequent, if not prevalent, constituents of the endodontic microbiome as reported by Korona‐Glowniak et al. (2021) and Martinho et al. (2021).

The PCA plot showed that PC1 distinguished the RC samples from saliva and plaque, that is, the RC samples can be characterized by fewer *Leptotrichia, Neisseria, Streptococcus*, (which had large positive loads), and more *Campylobacter* (which had large negative loads) in comparison with saliva and plaque. Both *Prevotella* and *Rothia* showed large positive loads on PC2, which discriminated saliva from plaque. The relative abundances of *Prevotella* and *Rothia* in saliva were 10.3% and 37.0%, respectively, against 0% and 1.2% in plaque. This finding is in accordance with that of a study by Simón‐Soro et al. (2013), where *Prevotella* and *Rothia* were present at very low levels or even absent in plaque despite showing considerable proportions in saliva.

In silico analysis using PICRUSt2 revealed that the nine most abundant Level 3 MetaCyc pathways in each sample were common despite their differences in community membership and structure. The top nine pathways that were commonly shared across the samples were proteinogenic amino acid biosynthesis, purine nucleotide biosynthesis, vitamin biosynthesis, fatty acid biosynthesis, sugar biosynthesis, phospholipid synthesis, cell wall synthesis, pyrimidine nucleotide biosynthesis, and 2′‐deoxyribonucleotide biosynthesis. These might be core pathways for oral microbiome that are essential and therefore stably abundant regardless of its microbial profiles. Among them, proteinogenic amino acid biosynthesis ranked first in all four samples with abundances ranging from 13.4% to 15.2%. Quinol and quinone biosynthesis ranked 10th in saliva, plaque, and RC42, but 35th in RC31, with abundances of 2.3, 2.9, 3.0, and 0.6%, respectively. The 10th pathway in RC31 was the fermentation of pyruvate with 2.0% abundance, which accounted for 1.7, 1.7, and 1.8% abundance and ranked 14th, 13th, and 12th in saliva, plaque, and RC42, respectively. Considering the small difference in its rank and ratio compared with those in other samples, pyruvate fermentation was not substantially elevated in RC31, and the decrease in quinol and quinone biosynthesis could be a characteristic of microbial function in RC31. The abundance of fatty acid biosynthesis was slightly different between RC and the other two samples. It ranked eighth in both RC samples but fourth in both saliva and plaque. The relative abundances of this pathway were 5.5, 6.1, 3.8, and 3.1% in saliva, plaque, RC42, and RC31, respectively. Further investigation with a larger sample is required to analyze whether this relatively lower level of fatty acid biosynthesis is widely shared among intracanal microbiomes.

The proportion of anaerobes seemed to affect the metabolic potential of the microbiome. In RC31, where anaerobic respiration was overrepresented at rank 21, anaerobes comprised 98.0% of the population. Its two major components, *Fusobacterium* and *Pseudoramibacter*, were both obligate anaerobes. In contrast, anaerobic respiration was barely observed in saliva and plaque, where facultative/obligate anaerobes formed 50.0% and 67.6% of the communities, respectively. Even in RC42, whose members were 89.0% anaerobes, anaerobic respiration was lower than aerobic respiration. This may indicate that anaerobic respiration within the microbial metabolism is mainly driven by obligate anaerobes, with little contribution from facultative anaerobes. As various quinones participate in respiratory electron transport, the dominance of obligate anaerobes in RC31 may explain the decrease in quinol and quinone biosynthesis accompanied by suppressed aerobic respiration. Anaerobes also seemed to enhance cobyrinate a,c‐diamide biosynthesis when they prevailed within the community. Cobyrinate a,c‐diamide biosynthesis is an early process in the anaerobic biosynthesis of adenosylcobalamin, and its rank and ratio in each sample were 69th (0.17%), 68th (0.16%), 45th (0.44%), and 30th (0.76%) in saliva, plaque, RC42, and RC31, respectively.

The clinical and radiographic findings of Teeth 42 and 31 were similar. Canal obturation was short of the apex (i.e., underfilled) in both cases, and radiolucent areas were observed at the apices. They had no sinus tracts but were sensitive to either percussion (Tooth 31) or palpation (Tooth 42). Both teeth were restored with a well‐fitted crown without apparent coronal leakage. Nevertheless, the membership and structure of the microbial communities were so distinctive that RC42 and RC31 plotted far from each other in PCA as well as in PCoA. Community profiling studies have revealed that the bacterial composition of the endodontic microbiota differs consistently among individuals with apical periodontitis. This indicates that apical periodontitis can result from multiple communities of different bacterial compositions. Therefore, the holistic concept that no single pathogen but rather the community per se is the unit of pathogenicity is now widely embraced (Siqueira & Rôças, 2005, 2009a, 2013). Our results showed that the bacterial profile of the RC could also vary within an individual, even if the conditions and symptoms of the involved teeth were nearly identical. As both teeth had a history of endodontic treatment, the previous intervention might have been responsible for the differences in their microbial profiles. The differences in treatment regimen or manner could have caused different bacterial species to survive within or accidentally infect the canals. However, an intraindividual comparison between primary infections is necessary to determine whether endodontic microbiomes can consistently vary against the same oral microbiome backdrop.

Furthermore, the two intracanal microbiomes showed sizeable differences in the abundance of several pathways by PICRUSt2, while the nine most abundant pathways were common. The higher level of glycolysis Ⅲ, that is, anaerobic glycolysis, in RC 31 was understandable, as its microbiome was conspicuously dominated by obligate anaerobes. ppGpp, the collective designation for the nucleotides guanosine tetraphosphate and guanosine pentaphosphate, is an indicator and regulator of the stringent response in bacteria (Irving et al., 2021; Ito et al., 2020; Srivatsan & Wang, 2008). ppGpp is synthesized from GTP and represses tRNA synthesis, thereby limiting the growth rate. preQ_0_, 7‐cyano‐7‐deazaguanine, is also synthesized from GTP, and is subsequently incorporated into certain tRNAs as hypermodified guanosine, called queuosine (McCarty et al., 2009). The elevated level of ppGpp metabolism in RC31 suggests that its microbiome is shutting down growth and priming survival under nutrient or energy starvation. This could explain the low level of preQ_0_ biosynthesis and low abundance of resource‐consuming pathways, such as CO_2_ fixation, lipopolysaccharide biosynthesis, and nitrogen compound metabolism, in RC31. For lipopolysaccharide biosynthesis, the proportion of Gram‐negative microbes can be a factor, too. Gram‐negative microbes accounted for 78.2% in RC42% and 55.4% in RC31. Fewer Gram‐negative microbes in RC31 could have resulted in a lower level of lipopolysaccharide biosynthesis. However, the difference in pathway abundance of this size, from 0.37% in glycolysis III to 0.86% in CO_2_ fixation, seemed to have no discernible effect on clinical manifestation in these two cases. Whether the abundances of these pathways influence clinical or histopathological findings in apical periodontitis needs further investigation using a more extensive collection of clinical samples obtained from multiple patients.

The limitation of this study is its small sample size, as it was conducted on only one patient. Considering the high interindividual diversity inherent in the oral microbiome, the taxonomic features found in this study might not reflect the general trend of the endodontic microbiomes. Future research with greater sample sizes including both primary/secondary infections, as well as symptomatic/asymptomatic cases is necessary to reveal the taxonomic and functional characteristics of the endodontic microbiomes.

## 5 CONCLUSIONS

The two intracanal microbiomes were more anaerobic, rich in Firmicutes, and scarce in Actinobacteriota and Proteobacteria, when compared with saliva and plaque. They were dissimilar despite their clinical and radiographic similarities, revealing intraindividual variability of the endodontic microbiome. The nine most abundant functional pathways were common to all the four samples although they had different microbial profiles. Further large‐scale investigations are necessary to understand the selective process to establish the endodontic microbiome within the oral cavity.

## AUTHOR CONTRIBUTIONS

Keiko Yamaki and Toru Tamahara were involved in conception, design, data collection, data analysis, data interpretation, drafting, and revising. Jumpei Washio, Takuichi Sato, Ritsuko Shimizu, and Satoru Yamada have contributed to conception, validation, data curation, and critical revising. All authors are in agreement with the manuscript.

## CONFLICT OF INTEREST STATEMENT

The authors declare no conflict of interest.

## ETHICS STATEMENT

This study was conducted according to the guidelines of the Declaration of Helsinki and approved by the Research Ethics Committee of Tohoku University Graduate School of Dentistry (No. 2019‐3‐1). Informed consent in a written form was obtained from the patient.

## Supporting information

Supporting information.

Supporting information.

Supporting information.

## Data Availability

The data that supports the findings of this study are available in the supplementary material of this article.
