# Supplementary figures and images for: Intracanal microbiome profiles of two apical periodontitis cases in one patient: A comparison with saliva and plaque profiles
Source: Clin Exp Dent Res. 2024 Mar 3;10(2):e862. doi: 10.1002/cre2.862 (PMC10909803; doi:10.1002/cre2.862)

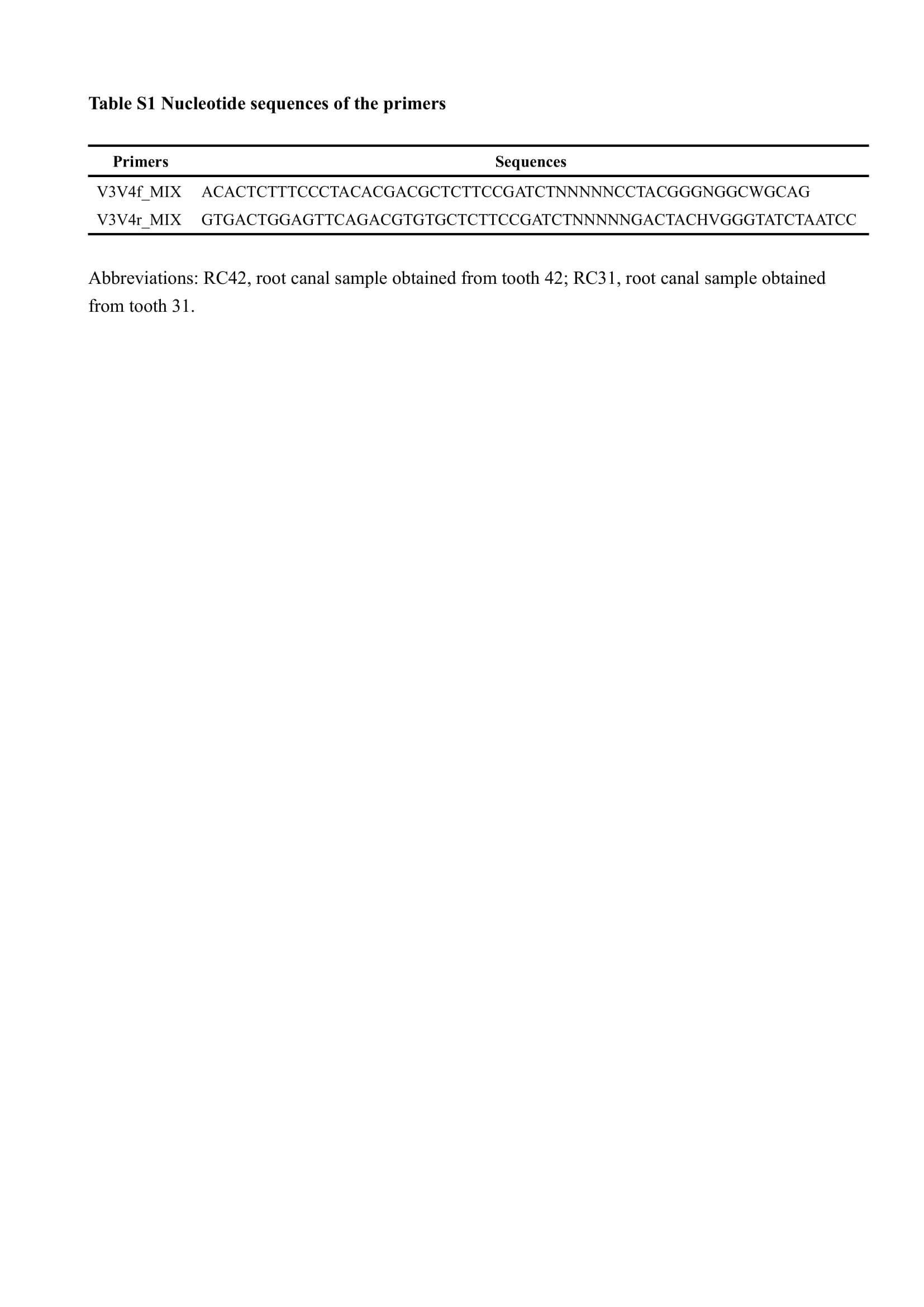

Supplement: Supplementary file 1 — Supporting information. [file CRE2-10-e862-s001.jpg]

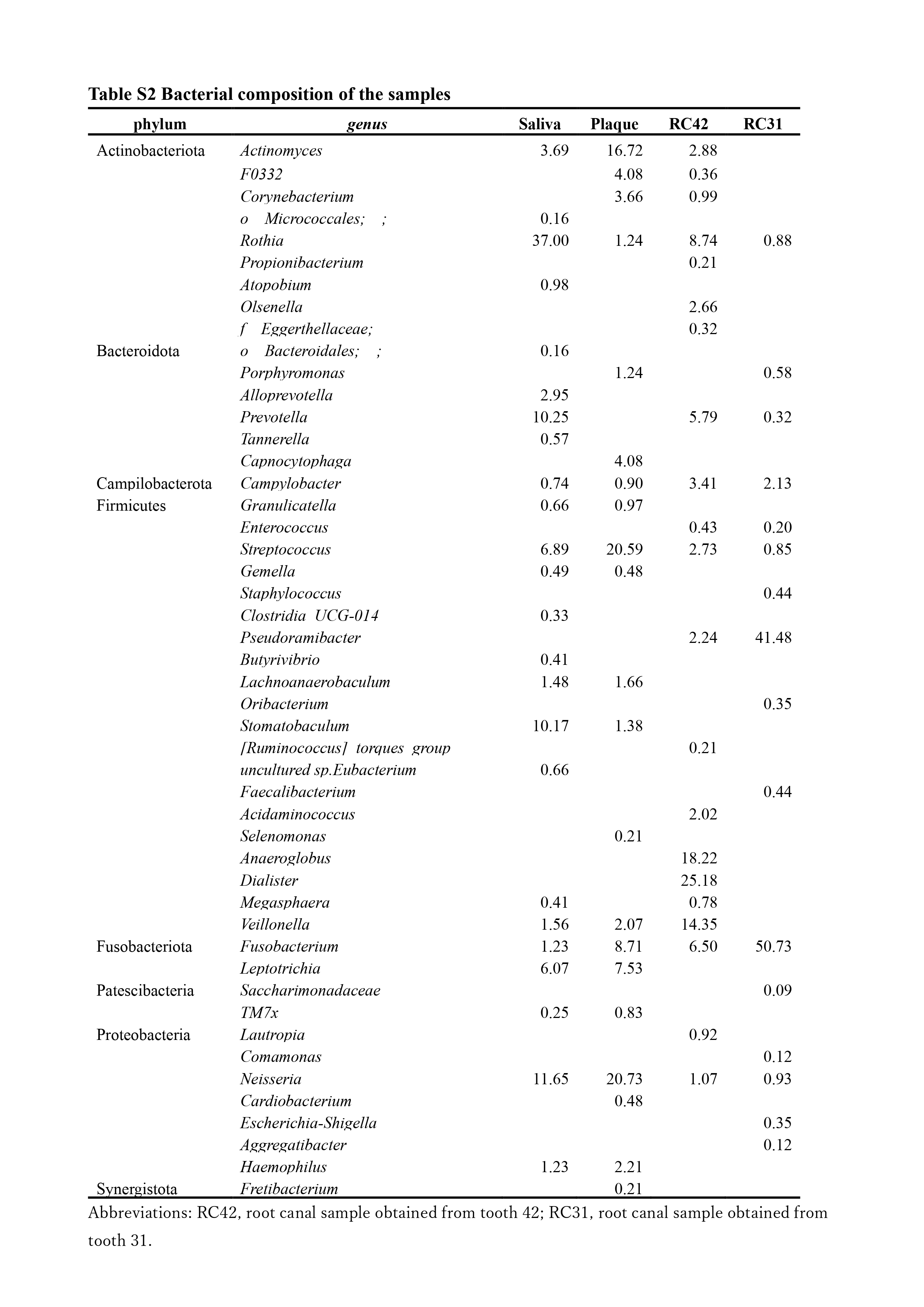

Supplement: Supplementary file 2 — Supporting information. [file CRE2-10-e862-s003.jpg]

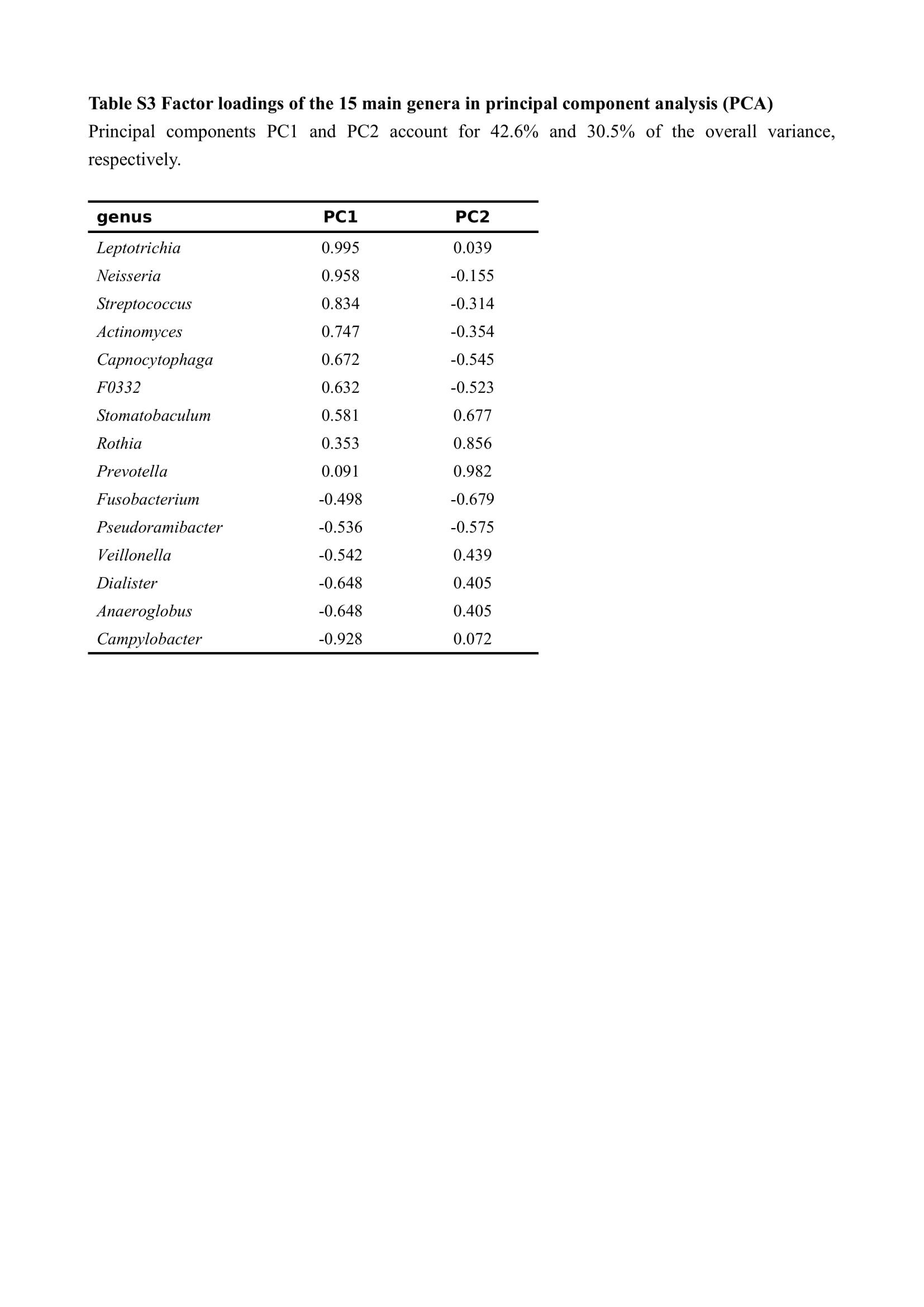

Supplement: Supplementary file 3 — Supporting information. [file CRE2-10-e862-s002.jpg]
